# Tardive Seizure Case Study

**DOI:** 10.1192/bjo.2026.11895

**Published:** 2026-06-30

**Authors:** Kazi Shams

**Affiliations:** Kent and Medway Mental Health NHS Trust, Maidstone, United Kingdom

## Abstract

**Aims:**

The patient is a 76 years old female without any past psychiatric history. She was married for 50 years and her husband died a year ago. Since then she became withdrawn and stopped eating, drinking. She was admitted to general hospital with hyponatremia.

**Methods:**

The patient was reviewed by Liaison psychiatry, she had symptoms of anhedonia, self-neglect, guilt, paranoia and psychomotor retardation. She was diagnosed with severe depressive episode with psychotic features and treated with Mirtazapine 45mg and Quetiapine 100mg.

There was no improvement on medication. She continued to decline food, water and had frequent hypoglycaemic attacks. Her BMI was 12.9. Bifrontal Electroconvulsive therapy (ECT) treatment was decided. During pre-ECT check-up her CRP was 38.

During the first episode, she didn’t have seizure on 10% stimulus. On 15% stimulus she had 109 seconds seizure on EEG. Post-ictal suppression was poor.

During the second episode, on 15% stimulus she had 77 seconds seizure on EEG.Post-ictal suppression was fair. Etomidate 10 mg was used as anaesthetic for both episodes.

While in recovery the patient was nonresponsive. Her blood sugar level was 4.6 mmol/L and she was given Dextrose. It rose to 9.9 mmol/L and she started to have seizure.

She had two observed seizures lasting 42 and 20 seconds. Her third seizure lasted 94 seconds while connected to EEG. She was given Propofol 20mg to stop the seizure.

She had a CT head without any acute findings. Chest X-ray showed shadow on the right base. Her CRP was 43 and sodium was 131.

**Results:**

Tardive seizure is a rare complication of ECT. It is a spontaneous seizure after ECT, there should be full recovery of consciousness before the onset.

Lansari et el (2025) found several factors associated with tardive seizure such as treatment that lowers the epileptogenic threshold, anaesthesia with Etomidate, prolonged seizure, and poor post-ictal suppression.

Another differential is that the seizure didn’t fully terminate after ECT, it became focal and later manifested as general tonic-clonic seizure. It is unclear if the patient fully recovered consciousness after ECT.

**Conclusion:**

The patient was started on Lamotrigine and remained seizure free. Due to her deteriorating physical health, she didn’t receive further ECT. There were some improvement to her mood and appetite with Mirtazapine and Venlafaxine.

Repeated ECT has been safe for many patients even after tardive seizure. Only 15% of patients developed further tardive seizure after restarting ECT (Warren et el 2022).

